# Live Imaging of Companion Cells and Sieve Elements in *Arabidopsis* Leaves

**DOI:** 10.1371/journal.pone.0118122

**Published:** 2015-02-25

**Authors:** Thibaud Cayla, Brigitte Batailler, Rozenn Le Hir, Frédéric Revers, James A. Anstead, Gary A. Thompson, Olivier Grandjean, Sylvie Dinant

**Affiliations:** 1 Institut Jean-Pierre Bourgin, INRA-AgroParisTech, UMR1318, ERL CNRS 3559, Saclay Plant Sciences, Versailles, France; 2 Univ. Bordeaux, CNRS, INSERM, Bordeaux Imaging Center, UMS 3420, Bordeaux, France; 3 INRA, Univ. Bordeaux, UMR1332 de Biologie du Fruit et Pathologie, Villenave d'Ornon, France; 4 College of Agricultural Sciences, The Pennsylvania State University, University Park, PA, United States of America; 5 Plateforme de Cytologie et Imagerie Végétale, UMR1318, INRA-AgroParisTech, Versailles, France; The Ohio State University, UNITED STATES

## Abstract

The phloem is a complex tissue composed of highly specialized cells with unique subcellular structures and a compact organization that is challenging to study *in vivo* at cellular resolution. We used confocal scanning laser microscopy and subcellular fluorescent markers in companion cells and sieve elements, for live imaging of the phloem in *Arabidopsis* leaves. This approach provided a simple framework for identifying phloem cell types unambiguously. It highlighted the compactness of the meshed network of organelles within companion cells. By contrast, within the sieve elements, unknown bodies were observed in association with the PP2-A1:GFP, GFP:RTM1 and RTM2:GFP markers at the cell periphery. The phloem lectin PP2-A1:GFP marker was found in the parietal ground matrix. Its location differed from that of the P-protein filaments, which were visualized with SEOR1:GFP and SEOR2:GFP. PP2-A1:GFP surrounded two types of bodies, one of which was identified as mitochondria. This location suggested that it was embedded within the sieve element clamps, specific structures that may fix the organelles to each another or to the plasma membrane in the sieve tubes. GFP:RTM1 was associated with a class of larger bodies, potentially corresponding to plastids. PP2-A1:GFP was soluble in the cytosol of immature sieve elements. The changes in its subcellular localization during differentiation provide an *in vivo* blueprint for monitoring this process. The subcellular features obtained with these companion cell and sieve element markers can be used as landmarks for exploring the organization and dynamics of phloem cells *in vivo*.

## Introduction

The cellular biology of phloem cells, especially the sieve element-companion cell complex (SE-CC), has been the focus of many studies in angiosperms, due to the key role of these cells in transporting metabolites, macromolecules and other signaling molecules [1]. During differentiation, sieve elements (SEs) and companion cells (CCs) undergo dramatic remodeling of their subcellular contents and organization. In the SEs, the nucleus, vacuole and cytoskeleton disintegrate, whereas the endoplasmic reticulum, mitochondria and plastids undergo significant spatial reorganization [2, 3]. These changes are accompanied by modifications to the subcellular organization of phloem parenchyma cells [4] and companion cells, which become highly metabolically active [4], with a dense cytoplasm containing mitochondria, enlarged vacuoles, rough endoplasmic reticulum and Golgi bodies [5]. Phloem cells also have specialized metabolic activities. For example, the hypoxia observed in phloem cells leads to specialized sugar metabolism in the companion cells, with the preferential use of sucrose synthases in sucrose metabolism, to conserve energy and oxygen [6–8], and the triggering of antioxidant defense systems in the sieve elements [9]. This accounts for the functional specialization of phloem parenchyma cells, companion cells and sieve elements [10, 11].

The correct identification of specific phloem cell types in different organs may be essential in comprehensive physiological studies. Phloem cell organization differs between organs. For example, the relative cell volumes of companion cells and sieve elements differ considerably between the collection, transport and release zones of the phloem [12]. The cells can be as small as a few microns in diameter. High-resolution transmission electron microscopy has provided detailed views of the organization and components of differentiating and mature sieve elements, companion cells and phloem parenchyma cells, in fixed phloem tissue [4]. However, the dynamic changes occurring in phloem cells can make it difficult to identify these cells by light microscopy [13].

In *Arabidopsis*, confocal laser scanning microscopy (CLSM) has successfully been used in the imaging of root phloem cells [14, 15], but it is difficult to obtain a sufficiently high resolution when imaging the phloem in leaves. This difficulty frequently leads to imprecise descriptions of the cellular and subcellular distributions of specific phloem cell components. In the leaf of Arabidopsis, the phloem is located 50 to 80 μm beneath the epidermal cells, and the diameter of sieve elements can be as small as 4 μm^2^ (1 μm X 4 μm or 2 μm X 2 μm) in minor veins [16].

In 1998, Knoblauch and van Bel conducted pioneering studies on imaging the functional phloem *in vivo*, based on confocal laser scanning microscopy of *Vicia faba* leaves, with the use of phloem-mobile fluorochromes to visualize mass flow [17]. This made it possible to characterize several phloem structures, including forisomes, and their dispersion in response to external and internal stimuli [18]. Unfortunately, fluorescent molecular tools for visualizing subcellular structures, such as GFP markers, are not available for use in *Vicia faba* phloem. The phloem peeling method [17] has been little used for other plant species, despite the higher degree of resolution that can be achieved. In this work, we applied this method to *Arabidopsis* leaves, and used fluorochromes and fluorescently labeled proteins to identify phloem cell types and subcellular compartments. A sufficiently high resolution was achieved for the formulation of simple criteria for unambiguous identification of the different cell types and for a detailed description of their subcellular organization *in vivo*. This approach was used to localize the phloem lectin PP2-A1 [19], the SEOR1 and SEOR2 filamentous proteins [14, 15] and two other sieve element proteins, the RTM1 (RESTRICTED TEV MOVEMENT 1) jacalin and the RTM2 (RESTRICTED TEV MOVEMENT 2) small heat shock protein, both of which restrict the long-distance movement of tobacco etch virus [20].

## Results

### *In vivo* observations of intact phloem in *Arabidopsis* leaves

**Fig 1:**
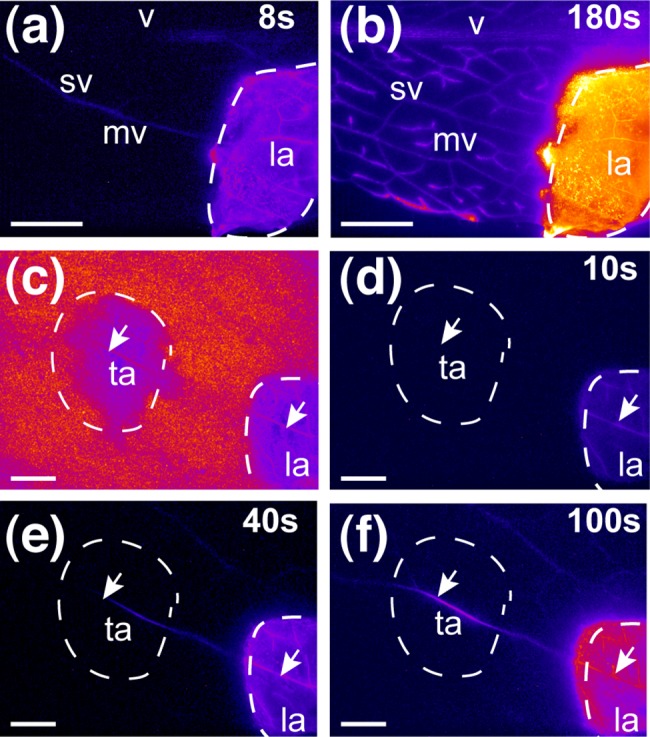
Assay of CFDA transport in the phloem cells of a detached *Arabidopsis* leaf. Leaves were observed by epifluorescence microscopy, under a fluorescent stereo microscope. The fluorescence appears as false color presentation using ImageJ’ ‘FIRE’ LUT. (a, b) Observation of fluorescence after the application of CFDA to a treated area of the leaf. (a) The veins in the loading area are already fluorescently labeled after 8 s. (b) After 180 s, the labeling extends to the main vein, secondary vein and minor veins around the loading area. (c-f) Observation of the transport of the fluorescent label after the application of CFDA to the treated area. (c) Observation of leaf autofluorescence immediately after application of the tracer. Treated areas are less fluorescent, enhancing observation of the vascular network (white arrows). For this experiment, the superficial layers were peeled away from a single vein in two areas: the loading area (la) and the transport area (ta). CFDA was loaded in the loading area. (d) 10 seconds after the application of CFDA. (e) 40 seconds after the application of CFDA, the fluorescence is transported and is visible in the transport area. (f) 100 seconds after the application of CFDA, the fluorescence has moved beyond the transport area. La: loading area; ta: transport area; mv: minor vein, sv: secondary vein; v: main vein. Scale bar = 5 mm.

We adapted the method described for *Vicia faba* [17], combining leaf peeling and light microscopy to view the vasculature of detached *Arabidopsis* leaves. This method yielded a higher resolution than could be obtained with untreated leaves. As sugar export capacity may decrease rapidly in *Arabidopsis* leaves following their excision from the plant [21], we investigated the possible impairment of phloem transport after the cutting of the petiole and peeling off of the leaf surface with a razor blade. We used the phloem symplasmic tracer 5,6 carboxyfluorescein-diacetate (CFDA) to investigate both phloem transport and sieve element integrity [22]. CFDA is a membrane-permeant dye that is cleaved by cellular esterase to release carboxyfluorescein (CF), a non membrane-permeant fluorescent form of the dye. Fluorescence rapidly progressed from the treated area into the veins (Fig. 1 A-B, S1 Movie), with CF reaching the main vein at an apparent velocity of 6–10 mm min^-1^, moving in a proximal direction toward the petiole of the detached leaf. This value was in the same range as the velocity determined in intact *Arabidopsis* plants (100 μm/s) [14], indicating that the treatment did not prevent phloem transport from the treated area to the petiole (i.e. sink-ward, as expected in intact leaves), and that leaf excision did not trigger the immediate sealing of the sieve tubes connected to the treated area. We also assessed the transport activity of the sieve tubes located immediately beneath the treated area. Two areas located 5 mm apart, close to the midrib, were peeled off, with one used for tracer application and the other, in a more proximal position, used for observations of tracer translocation (Fig. 1 C-F). After loading in the acropetal area, the tracer moved basipetally across the second area on its way to the main vein, at a flow velocity of up to 10 mm min^-1^ (Fig. 1 D-F). The fluorescence continued to progress along the veins in this observation area, with minimal fluorescence detectable outside the veins, indicating that the sieve elements were continuing to translocate material beneath the treated area. Thus, the treatment of abaxial tissues from a detached *Arabidopsis* leaf does not impede phloem transport function, at least during the first few minutes after cutting. Phloem imaging is, therefore, possible.

### Use of the PP2-A1:GFP fusion protein as a marker of companion cells

**Fig 2:**
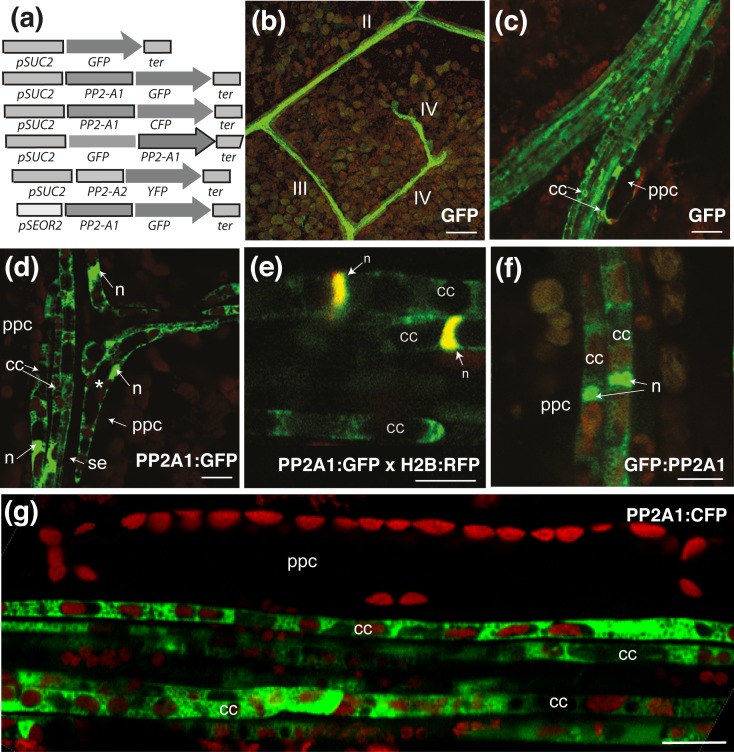
Imaging of PP2-A1:GFP and free GFP in the phloem cells of a four-week-old leaf. Visualization of soluble GFP or PP2-A1 tagged with GFP or CFP in leaves from *pSUC2*:*PP2-A1*:*GFP, pSUC2*:*PP2-A1*:*GFP, pSUC2*:*PP2-A1*:*CFP* and *pSUC2*:*GFP* plants. Images were obtained by CLSM and fluorescence is shown in false colors. GFP or CFP signals are shown in green, and chlorophyll autofluorescence is shown in red. GFP-tagged PP2-A1 is found in the cytosol and nucleus of the companion cells. (a) Constructs for the imaging of fluorescence-tagged PP2-A1 and PP2-A2. (b) Observation, in a treated leaf area, of soluble GFP produced under the control of the *SUC2* promoter, in a *pSUC2*:*GFP* plant. GFP fluorescence is observed in veins of all orders (II, III and IV). (c) Localization of GFP fluorescence in a main vein from a *pSUC2*:*GFP* plant. The soluble GFP is found in companion cells and phloem parenchyma cells. (d) Localization of PP2-A1:GFP fluorescence to the junction of two veins from a *pSUC2*:*PP2-A1*:*GFP* plant. Bent companion cells (indicated by *) are typically found at such junctions. (e) Localization of PP2-A1:GFP in a vein from a *pSUC2*:*PP2-A1*:*GFP* plant. GFP is found only in the companion cells. This observation was made on a cross between the *pSUC2*:*PP2-A1*:*GFP* (in green) and *p35S*:*H2B*:*RFP* lines (in color red), so the nucleus is shown in yellow in the overlay, due to H2B:RFP and PP2-A1:GFP fluorescence. (f) Localization of GFP:PP2-A1 fluorescence in a minor vein from a *pSUC2*:*GFP*:*PP2-A1* plant. (g) Localization of *PP2-A1*:*CFP* fluorescence (shown in green) in a minor vein from a *pSUC2*:*PP2-A1*:*CFP* plant. Typical distribution of plastids in the companion cells is observed. In the companion cells, the autofluorescent chloroplasts were aligned in a single file, whereas, in phloem parenchyma cells, the chloroplasts were located at the cell periphery. cc = companion cell; ppc = phloem parenchyma cell; se: sieve element. n: nucleus. Scale bar = b) 50 μm; (c)–(g) 10 μm.

Various transgenic *Arabidopsis* Col-O lines producing green fluorescent protein (GFP) or other fluorescent proteins (CFP or YFP) were generated for live imaging of the phloem (Table 1). Genes encoding free, soluble GFP or GFP fused to the phloem lectin PP2-A1 [19] were expressed under the control of a companion cell-specific promoter (Fig. 2 A, S1 Table). The *SUC2* promoter [23] was used in all constructs in preference to the *PP2-A1* promoter [19], which frequently drives cosuppression of the endogenous *PP2-A1* gene (unpublished results). The veins were observed *in vivo* by confocal laser scanning microscopy (CLSM), immediately after leaf peeling. Fluorescence was bright in veins of the various orders (i.e. main, secondary and minor; Fig. 2 B). Longitudinal observations of the phloem cells were carried out. In *pSUC2*:*PP2-A1*:*GFP* plants, fluorescence was observed in the cytosol and nucleus of companion cells, with no fluorescence in the sieve elements or phloem parenchyma cells (PPCs) (Fig. 2 D,E). This subcellular distribution was identical to that in undetached leaves, as shown by *in vivo* observations of leaves *in planta* using stereo fluorescence microscope (S1 Fig.). Similar findings were recorded for all veins with the *pSUC2*:*GFP*:*PP2-A1* and *pSUC2*:*PP2-A1*:*CFP* constructs (Fig. 2 F-G). In an individual confocal section, up to 20 companion cells could be observed in the main veins, whereas three to five companion cells were observed in the smallest minor veins. Similar images were obtained with the YFP-tagged PP2-A2, a phloem lectin closely related to PP2-A1 [19], in *pSUC2*:*PP2-A2*:*YFP* plants (S2 Fig., A). By contrast, free GFP fluorescence was observed in both phloem parenchyma cells and companion cells in *pSUC2*:*GFP* plants (Fig. 2 C). As the promoter sequence used was the same as that used for PP2 fusions, for which no signal was detected in phloem parenchyma cells, these findings demonstrate the high specificity of this promoter for companion cells, and they indicate that the soluble GFP was transported from the companion cell into the phloem parenchyma cells via the plasmodesmata. Phloem parenchyma cells were larger (up to 17 μm in diameter) and were found in lateral positions within the veins. By contrast, PP2 fusions, which have an apparent molecular weight (MW_app_) of 55 and 45 kDa for the PP2-A1 and PP2-A2 fusions, respectively, did not diffuse from the companion cells to the phloem parenchyma cells, unlike free GFP (MW_app_ of 27 kDa), consistent with the plasmodesmata having a size exclusion limit for nonspecific trafficking of between 30 and 40 kDa in *Arabidopsis* leaves [24].

**Table 1:** Phloem markers available for the identification of phloem cells.

Constructs	Cell type	Description (MW_app_ of the GFP fusion)	AGI number (name of the protein)
*pSUC2*:*PP2-A1*:*GFP* *pSUC2*:*PP2-A1*:*CFP* *pSUC2*:*GFP*:*PP2-A1*	Companion cell	Nucleus and cytosol (55 kDa)	At4g19840 (PP2 phloem lectin)
*pSUC2*:*PP2-A2*:*GFP*	Companion cells	Nucleus and cytosol (45 kDa)	At4g19850 (PP2 phloem lectin)
*pSEOR2*:*PP2-A1*:*GFP*	Sieve element	Around mitochondria and SE bodies (≈ 1 μm) (55 kDa)	At4g19840 (PP2 phloem lectin)
*pSEOR1*:*SEOR1*:*GFP*	Sieve element	P-proteins (112 kDa)	At3g01680 (P-protein)
*pSEOR2*:*SEOR2*:*GFP*	Sieve element	P-proteins (121 kDa)	At3g01670 (P-protein)
*pRTM1*:*GFP*:*RTM1*	Sieve element	Around SE bodies (≈ 1 μm) (46 kDa)	At1g05760 (RESTRICTED TEV MOVEMENT 1; jacalin)
*pRTM2*:*RTM2*:*GFP*	Sieve element	Bodies at the periphery of the SE (68 kDa)	At5g04890 (RESTRICTED TEV MOVEMENT 2; sHSP)

Moreover, no fluorescence was observed in the sieve elements with GFP and PP2 fusions, because of rapid translocation towards sink organs, dilution in the cytosol of the sieve element, or, for PP2 fusions, an inability of the fluorescent proteins to pass through pore-plasmodesmata units (PPUs).

### Cytological criteria for phloem cell identification

In *pSUC2*:*PP2-A1*:*GFP and pSUC2*:*PP2-A1*:*CFP* plants, in which the fluorescent signal was strictly limited to companion cells, we analyzed in more detail the subcellular organization of companion cells. The elongated shape and dimensions of the companion cells (mean diameter of 4 μm and length of up to 70–100 μm) labeled with the fluorescent protein were easily discerned, except at the vein junctions, where curved companion cells were characteristically enlarged at the bend (Fig. 2 D). Assessments of chlorophyll autofluorescence showed a remarkable alignment of chloroplasts in single file, in successive companion cells (Fig. 2 G). Up to 10 chloroplasts could be observed in individual confocal sections. The chloroplasts occupied most of the diameter of the cells. This feature was sufficiently characteristic to constitute a hallmark of companion cells, because no other cell type displayed such an alignment of chloroplasts. In most companion cells, one chloroplast was found adjacent to one side of the cell nucleus. The mean diameter of the chloroplasts in companion cells was 3.21 ± SD 0.66 μm (*n* = 662), smaller than that in phloem parenchyma cells (4.08 ± SD 0.99 μm; *n* = 278) and mesophyll cells (5.58 ± SD 1.01 μm; *n* = 250) (S3 Fig.). Other cell types also displayed markedly different chloroplast organizations. For example, in phloem parenchyma cells, the chloroplasts were arranged at the cell periphery, mostly on the side opposite the companion cell (Fig. 2 G; S2 Movie).

### Subcellular organization of companion cells

**Fig 3:**
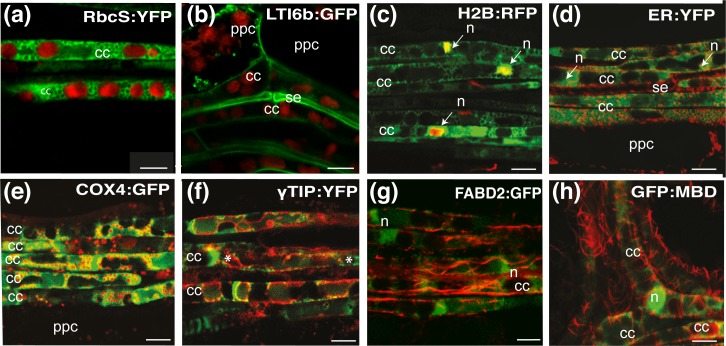
Imaging of subcellular compartments in companion cells and phloem parenchyma cells. (a, c-h) Fluorescent proteins observed in leaves from plants resulting from crosses between *pSUC2*:*PP2-A1*:*CFP* plants and lines carrying fluorescent proteins targeted to different subcellular compartments. Overlay images, obtained by CLSM, with fluorescence signals shown in false colors; CFP is shown in green (a, c-h) and GFP, YFP or RFP in red (a, c-h) except in (b). Superimposed pixels are shown in yellow. (a) Observation of chloroplasts in the companion cells of a *pSUC2*:*PP2-A1*:*CFP* x *p35S*:*RbcS*:*YFP* plant (PP2-A1:CFP fluorescence is shown in green and RbcS:YFP in red). (b) Observation of the typical morphology of phloem cells of a *p35S*:*GFP*:*LTI6b* plant. GFP is shown in green and chloroplast autofluorescence is shown in red. Two types of phloem cells—companion cells and phloem parenchyma cells—were identified on the basis of plastid distribution (autofluorescence, shown in red). In these plants, GFP:LTI6b fluorescence (in green) could also be used to identify sieve elements. Phloem parenchyma cells are the largest cells and are located on the edge of the vasculature. Sieve elements lack chloroplasts. Companion cells display typical chloroplast alignments. (c) Observation of nuclei in the companion cells of a *pSUC2*:*PP2-A1*:*CFP* x *p35S*:*H2B*:*RFP* plant. Companion cells have square-like nuclei. (d) Observation of the endoplasmic reticulum in the companion cells of a *pSUC2*:*PP2-A1*:*CFP* x *p35S*:*ER*:*YFP* plant. In companion cells, the ER is found principally next to the plasma membrane and around the nucleus. On this image, the ER can also be seen in a sieve element aligned between two arrays of companion cells. (e) Observation of mitochondria in the companion cells of a *pSUC2*:*PP2-A1*:*CFP* x *p35S*:*COX4*:*GFP* plant. On this image, large numbers of mitochondria can be seen in the companion cells. (f) Observation of vacuoles in the companion cells of a *pSUC2*:*PP2-A1*:*CFP x p35S*:*yTIP*:*YFP* plant. The image shows several vacuoles per companion cell. (g) Observation of actin network in the companion cells of a *pSUC2*:*PP2-A1*:*CFP* x *p35S*:*FABD2*:*GFP* plant. Thick actin bundles can be seen whereas thin actin filaments are barely detectable. (h) Observation of cortical microtubules in bent companion cells at a vein junction in a *pSUC2*:*PP2-A1*:*CFP* x *p35S*:*GFP*:*MBD* plant. Stars indicate the vacuoles. Scale bar = 5 μm.

Companion cells are highly compartmentalized (Fig. 2 G). Their subcellular organization was analyzed in crosses between the *pSUC2*:*PP2-A1*:*CFP* line and transgenic lines producing GFP or YFP fluorescent marker molecules (S2 Table) (Fig. 3, S4 Fig.). These cells could be identified on the basis of chloroplast alignment or CFP fluorescence from the *PP2-A1*:*CFP* fusion (Fig. 3 A). An example of this approach is provided by the analysis of a cross with the *p35S*:*GFP*:*LTI6b* line [25], which carries a plasma membrane marker. Fluorescence was readily observed at the plasma membrane (PM) in companion cells (Fig. 3 B). In *p35S*:*H2B*:*RFP* plants expressing the fluorescent histone 2B (H2B:RFP) nuclear marker, the nuclei of companion cells appeared dense and compacted (Fig. 3 C). Similarly, large numbers of mitochondria (Fig. 3 E; S2 Fig., B; S2 Movie) could be seen in the cytoplasm of the companion cells, along with the endoplasmic reticulum (Fig. 3 D). There were also numerous vacuoles (Fig. 3 F), with one vacuole often in contact with the nucleus, in addition to chloroplasts (Fig. 3 A). These results were obtained with plants expressing an endoplasmic reticulum marker (ER:YFP), the cytochrome *c* oxidase IV mitochondrial marker (COX4:YFP), the vacuolar aquaporin membrane marker (γTIP:YFP) and the RuBisCO marker (RbcS:YFP), respectively. We also observed the organization of the cytoskeleton in plants expressing an actin filament marker (fABD2:GFP) or a cortical microtubule marker (GFP:MBD) (Fig. 3 G-H). The organization of the actin filaments was difficult to determine with fABD2:GFP, because the fluorescence signal was weaker than for other markers (Fig. 3 G). A few thick bundles of actin filaments were observed at the cell periphery, mostly aligned along the long axis of the cell. Individual fine actin filaments were difficult to visualize. As observed in GFP:MBD plants, the cortical microtubules (CMTs) were aligned in companion cells, as in other cell types, in a transverse or oblique orientation, although heterogeneous arrays could be observed in some cells (Fig. 3 H, S2 Fig., C-E).

### Subcellular organization of the sieve elements

**Fig 4:**
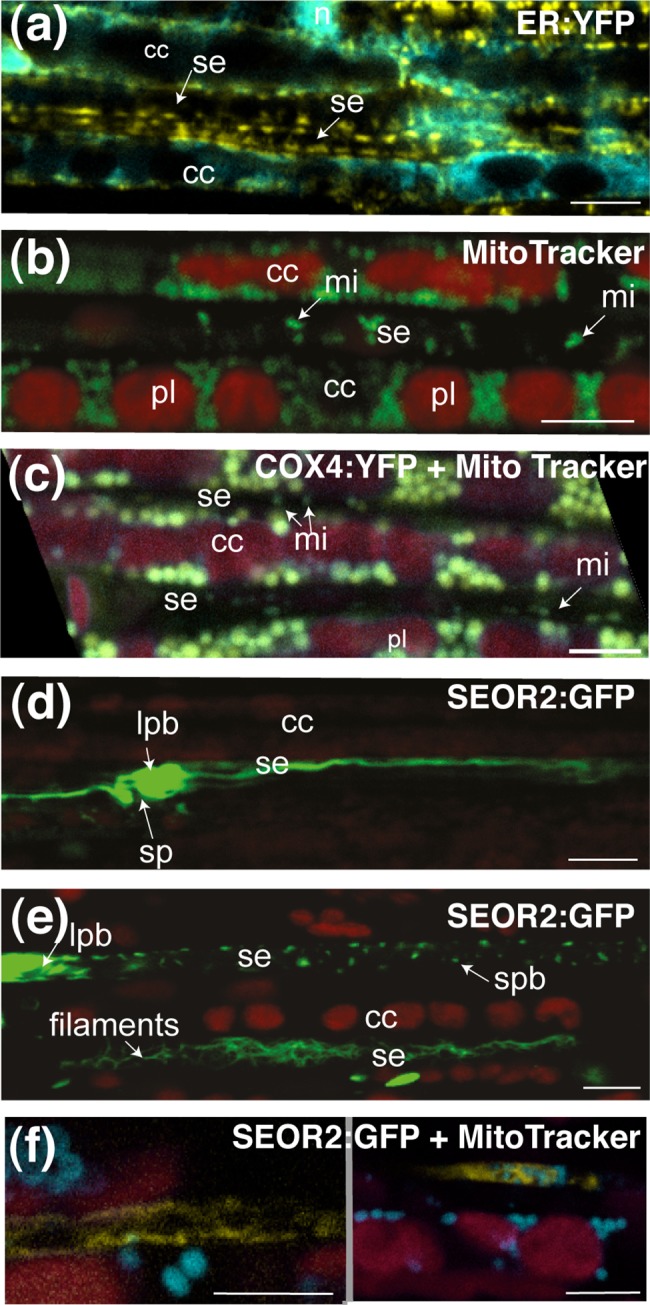
Imaging of subcellular compartments and known protein bodies in sieve elements. Fluorescent proteins observed in leaves from plants carrying GFP expressed in different subcellular compartments. Images were obtained by CLSM. Fluorescence is shown in false color. (a) Observation of the endoplasmic reticulum in the companion cells and the sieve elements of a *p35S*:*ER*:*YFP* x *pSUC2*:*PP2-A1*:*GFP* plant (same section as in Fig. 3D). YFP fluorescence is shown in yellow and GFP is shown in blue. (b) Observation of mitochondria with MitoTracker fluorescent dye, presented in false colors (green). Plastid autofluorescence is shown in red. Mitochondria are found in both the companion cells and sieve elements. (c) Observation of mitochondria with MitoTracker fluorescent dye, presented in false colors (blue) in the phloem of *p35S*:*COX4*:*GFP* plant (in yellow). Plastid autofluorescence is shown in red. Co-labeling of mitochondria with MitoTracker and COX4:GFP, in both the companion cells and sieve elements, is shown in green. Arrows indicate mitochondria in the sieve elements.(d), (e) and (f) Observation of the P-proteins in the sieve elements of a *pSEOR2*:*SEOR2*:*GFP* plant. In (d) and (e), GFP fluorescence is shown in green, plastid autofluorescence in red. In these images, the P-proteins form a typical plug (arrow) next to the sieve plate (in (c)), and discrete filaments in the lumen of the sieve element and protein agglomerates (in (d)). In (f), GFP fluorescence is shown in yellow, plastid autofluorescence in red, and MitoTracker fluorescence in blue.

The subcellular organization of sieve elements was also examined. The *GFP*:*LTI6b* marker labeled the plasma membrane in the sieve elements (1–3 μm diameter in minor veins and 3–4 μm in larger veins), with brighter fluorescence was observed in the vicinity of the sieve plates (Fig. 3 B). Plants expressing the ER marker (ER:YFP) displayed a succession of fine fluorescent stacks at lateral positions in sieve elements (Fig. 4 A). For the mitochondrial marker (COX4:YFP), we observed faint fluorescence colocalized with the vital dye MitoTracker Red (Fig. 4 B,C), indicating this marker is active in sieve elements. Other fluorescent GFP markers were more difficult to visualize. For the nuclear marker (H2B:RFP) or the tonoplast marker (γTIP:YFP), the breakdown of the nucleus and tonoplast during differentiation of the sieve elements accounted for the absence of fluorescence. For the actin marker (fABD2:GFP), the microtubule marker (GFP:MBD) and the chloroplast marker (RbcS:YFP), no fluorescence signal was detected in the sieve elements. Sieve element plastids are not photosynthetic, and RuBisCO is probably degraded during sieve element differentiation. The actin cytoskeleton has been identified in sieve elements [14, 26], and the lack of fluorescence may be due to the signal being below the detection threshold, or the GFP and YFP fusions being unable to pass through the PPUs between the companion cells and sieve elements.

We also examined the subcellular organization of sieve tubes in transgenic *Arabidopsis* lines producing GFP-tagged sieve element proteins (Table 1, S2 Table). As expected, filaments of up to 200 μm long and resembling those described in roots *in planta* [14, 15] were observed in the sieve elements (Fig. 4 D-F) of the leaves of *pSEOR2*:*SEOR2*:*GFP* plants producing a GFP-labeled structural phloem filament protein, SEOR2 [15]. Similar findings were obtained for undetached leaves observed *in planta* using stereo fluorescence microscope (S1 Fig., C). These filaments were not colocalized with mitochondria (Fig. 4 E,F). In some cells, the protein appeared to accumulate as spherical protein agglomerates similar to those reported in immature sieve elements in roots [14, 15]. Similar images were obtained for plants expressing the SEOR1:GFP fusion. We then investigated the sieve tube proteins RTM1 and RTM2.

**Fig 5:**
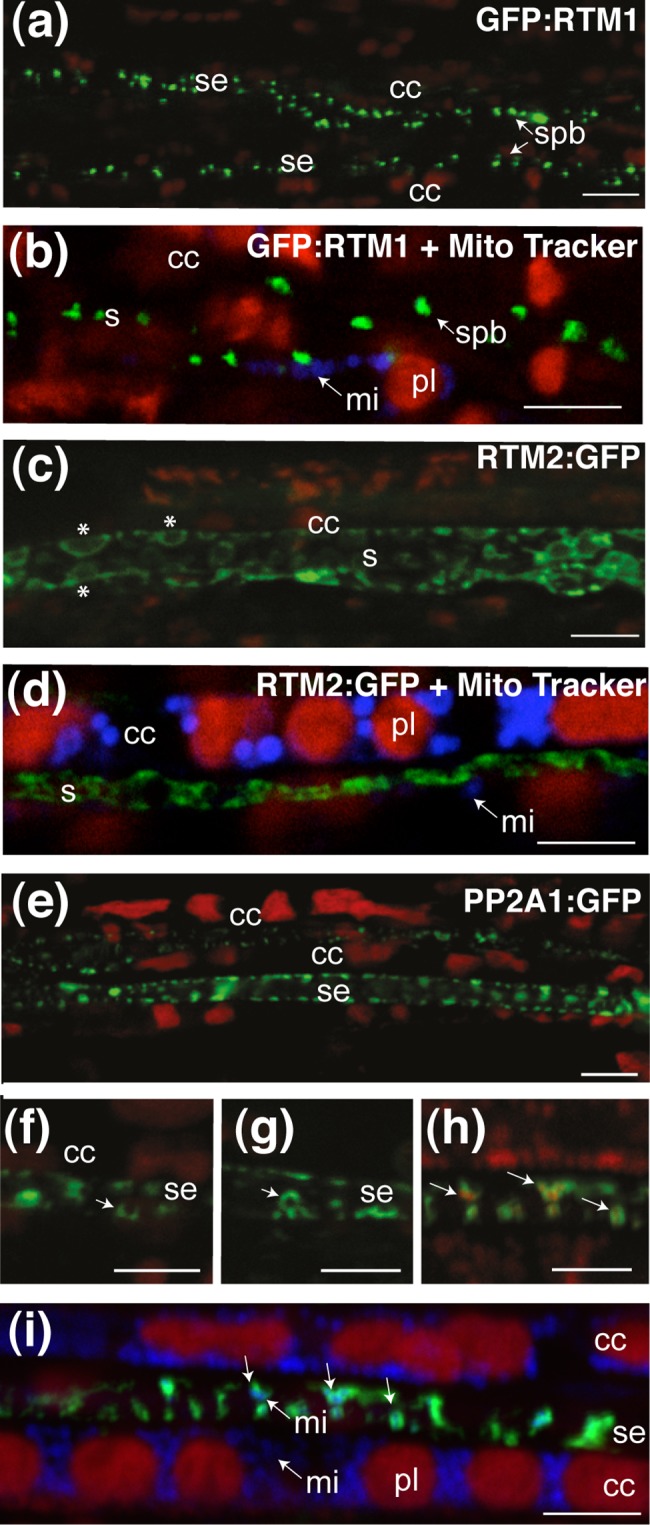
Imaging of subcellular compartments and new markers in sieve elements. Fluorescent proteins observed in leaves from plants carrying GFP expressed in different subcellular compartments. Images were obtained by CLSM. Fluorescence is shown in false color. (a) and (b) Observation of discrete bodies in a *pRTM1*:*GFP*:*RTM1* plant. The bodies in the sieve elements are up to 1 μm in diameter. In (h) colocalization of GFP:RTM1 with MitoTracker. GFP fluorescence is shown in green, plastid autofluorescence in red, and MitoTracker in blue. (c) and (d) Observation of fluorescence in a *pRTM2*:*RTM2*:*GFP* plant. The signal is mostly localized at the vicinity of the plasma membrane, within the sieve element. It is also observed around spherical bodies (*) located next to the plasma membrane. In (d) colocalization of RTM2:GFP with MitoTracker. GFP fluorescence is shown in green, plastid autofluorescence in red, and MitoTracker in blue. (e)–(i) Observation of PP2-A1:GFP in a *pSEOR2*:*PP2-A1*:*GFP* plant. PP2-A1 fluorescence indicates the presence of PP2-A1 in discrete spots located at the borders of the cell and in the vicinity of the sieve plate. In (f) and (g), details of the localization of PP2-A1:GFP around organelles present in the sieve elements. Arrows indicates circular structures surrounded by PP2-A1. In (h) colocalization of PP2-A1:GFP with MitoTracker Red. GFP fluorescence is shown in green and MitoTracker fluorescence is shown in red. In (i) colocalization of PP2-A1:GFP with mitochondria. GFP fluorescence is shown in green, chloroplast autofluorescence in red and MitoTracker fluorescence is shown in blue. Arrows indicate mitochondria surrounded by PP2-A1:GFP. mi: mitochondrion. pl: chloroplast. sp: sieve plate. lpb: large protein body. spb: small protein body. Scale bar = 5 μm.

**Fig 6:**
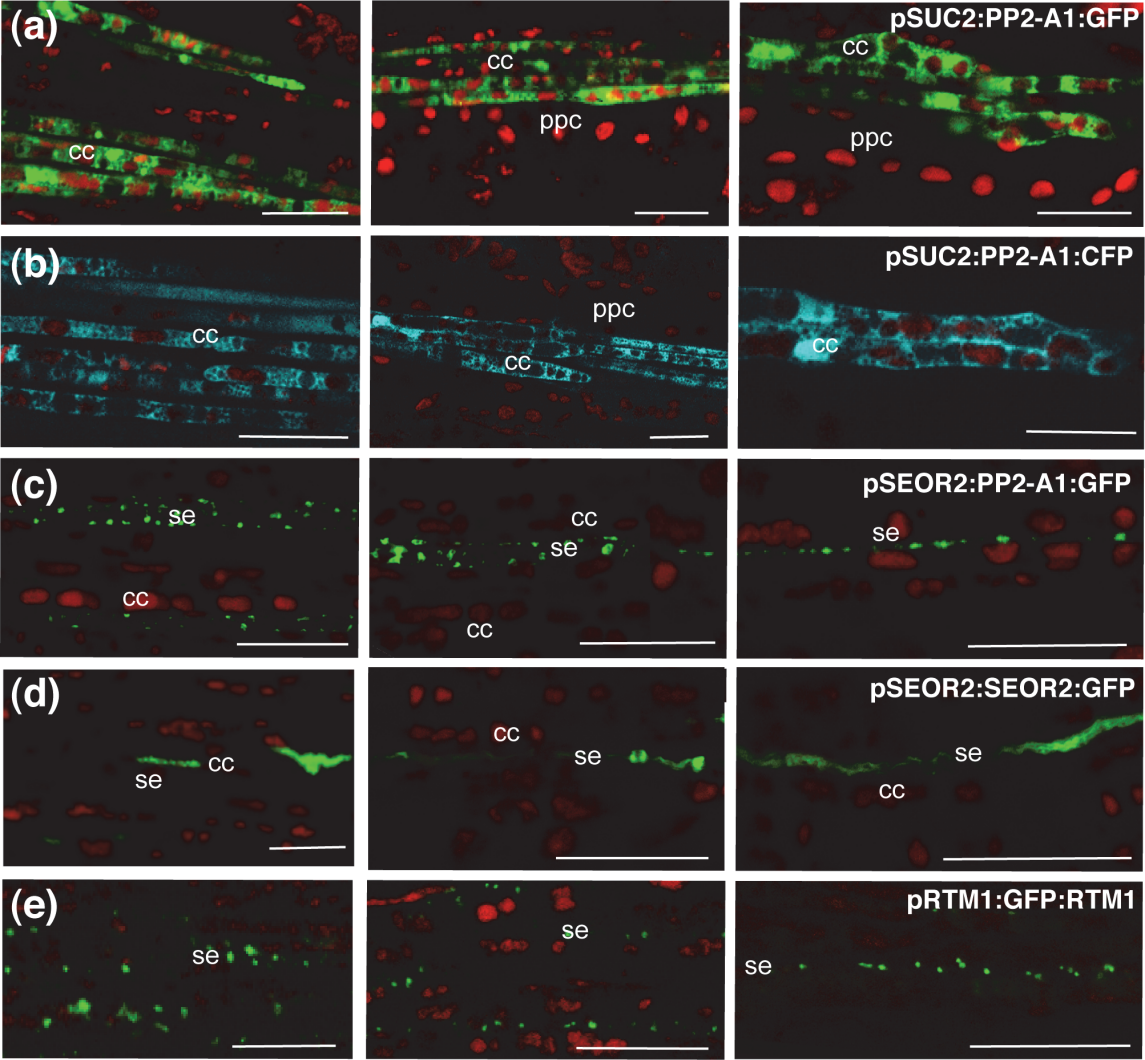
Imaging of phloem markers in all orders of veins. Fluorescent proteins observed in leaves from plants carrying GFP or CFP fused to different proteins, either in the companion cells, or in the sieve elements. Images were obtained by CLSM and luorescence is shown in false color, with GFP shown in green, CFP shown in blue and plastid autofluorescence shown in red. Left panel: main vein. Middle panel: secondary vein. Right panel: minor vein. (a) Observation of *pSUC2*:*PP2-A1*:*GFP* plant. (b) Observation of *pSUC2*:*PP2-A1*:*CFP* plant. (c) Observation of *pSEOR2*:*PP2-A1*:*GFP* plant. (d) Observation of *pSEOR2*:*SEOR2*:*GFP* plant. (e) Observation of *pRTM1*:*GFP*:*RTM1* plant. Scale bar = 20 μm.

We analyzed the lines expressing the *pRTM1*:*GFP*:*RTM1* and *pRTM2*:*RTM2-GFP* constructs in the TEV-susceptible C24 (*rtm1/rtm1*) and Col-O *rtm2-1* mutant (*rtm2/rtm2*) background (Table 1), in which they provided functional complementation for TEV restriction with RTM1 [20, 27] and for LMV restriction with RMT2 (Revers, personal communication). In *pRTM1*:*GFP*:*RTM1* plants, an alignment of spherical protein bodies with an apparent diameter of 1.16 ± 0.01 μm (*n* = 612) was found at the periphery of individual sieve elements (Fig. 5 A), with up to 30–40 bodies in the sieve elements found in the larger veins. These bodies were not colocalized with mitochondria (Fig. 5 B). Their dimensions were of a similar range to that of sieve element plastids [14, 28, 29]. By contrast in *pRTM2*:*RTM2*:*GFP* plants, a reticulate organization lining the plasma membrane and surrounding bodies smaller than mitochondria was observed in the sieve elements (Fig. 5 C,D). The nature of these bodies remains unknown, but structures of a similar dimension were observed in transmission electron micrographs of sieve elements in *Arabidopsis* leaves (S5 Fig.). In the lines expressing these sieve element fluorescent markers, as lines expressing companion fluorescent markers, similar findings were obtained for all orders of veins, other than for RTM2, which was difficult to image in the minor veins (Fig. 6).

### Localization of PP2-A1 in the sieve elements

PP2 phloem lectins were once considered to P-protein components [30], but their localization in the sieve elements remains a matter of debate [31]. Immunolocalization studies have shown that PP2-A1 accumulates in small bodies in the sieve elements [31]. In the *pSEOR2*:*PP2-A1*:*GFP* lines, in which expression of the GFP fusion is driven by the sieve element-specific SEOR2 promoter [15, 32], fluorescence was detected around bodies at the lateral cell borders of the sieve elements (Fig. 5 E-I). These bodies had a bimodal size distribution, with a main peak for their longest dimension at 0.52 μm and a second, less prominent peak at 0.96 μm, accounting for about one fifth of the bodies (*n* = 328) (S6 Fig., G). They were motionless, indicating they were anchored to membranes. The GFP fluorescence signal could be mostly observed as small rings surrounding an unlabeled area (Fig. 5 F-G, S6 Fig., A-F), showing that the GFP-tagged proteins accumulated principally at the periphery of some organelles. In *pSEOR2*:*PP2-A1*:*GFP* leaves stained with MitoTracker Red, the fluorescence was surrounded by GFP fluorescence (Fig. 5 H,I), demonstrating that the class of PP2-A1-GFP-labeled bodies with a small diameter (0.52 μm) corresponded to sieve element mitochondria. Transmission electron micrographs of sieve elements confirmed that the mitochondria were aligned along the plasma membranes, with an apparent diameter of 0.458 ± 0.026 μm (*n* = 12) (S6 Fig., H-J). The larger bodies labeled by PP2-A1:GFP (mean diameter of 0.96 μm) did not colocalize with MitoTracker Red.

### Distribution of PP2-A1:GFP in differentiating sieve elements

**Fig 7:**
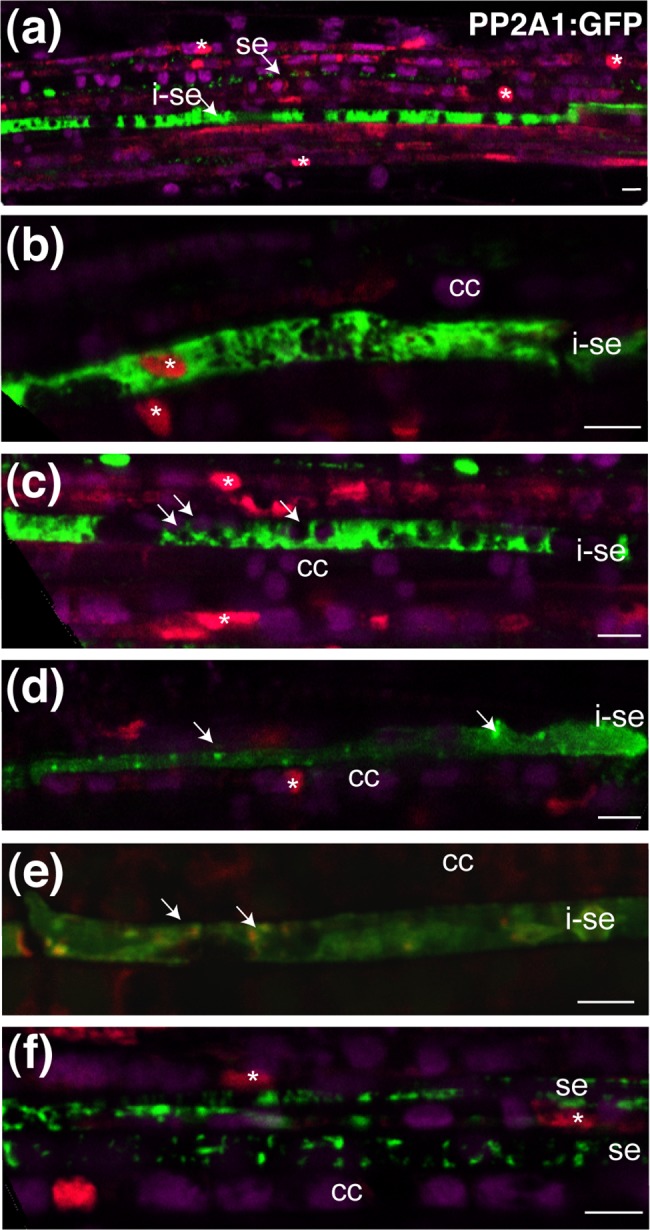
Imaging of PP2-A1:GFP in sieve elements undergoing differentiation. Fluorescent PP2-A1:GFP protein observed in leaves of *pSEOR2*:*PP2-A1*:*GFP* plants. Images were obtained by CLSM. GFP fluorescence is shown in false color green, propidium iodide in nuclei is shown in red and plastid autofluorescence in magenta except in (e). (a) General overview of a main vein showing mature and immature sieve elements. (b) Immature sieve element with soluble PP2-A1:GFP. In this cell, a number of subcellular compartments, including organelles and the nucleus, are still present. (c) Immature sieve element with soluble PP2-A1:GFP, in which the nucleus is no longer observed, although plastid autofluorescence is still present. Arrows indicate plastids in an immature sieve element. (d) In some cells that have begun to differentiate, PP2-A1:GFP fluorescence aggregates around small organelles. As sieve elements mature, their organelles gradually disappear. PP2-A1:GFP is present in an aggregated form around organelles, presumably mitochondria, although still present in a soluble form in the cytosol. Arrows indicate dense PP2-A1 material, presumably around mitochondria and plastids. (e) In sieve element ongoing differentiation, PP2-A1:GFP fluorescence, shown in green, aggregates around mitochondria stained with MitoTracker (shown in red). Arrows indicate mitochondria. (f) In mature sieve elements, PP2-A1:GFP is present only attached to organelles. * indicates nuclei. Se: sieve element; i-se: immature sieve element; companion cell. Scale bar = 5 μm.

The *SEOR2* promoter is active in immature sieve elements [32], making it possible to localize PP2-A1:GFP in differentiating sieve elements. In *pSEOR2*:*PP2-A1*:*GFP* plants, differentiating sieve elements were readily recognizable on the basis of their bright fluorescence signal. Differentiation could be monitored by staining nuclei with propidium iodide and recording plastid autofluorescence. At early stages of differentiation in cells that still contained a nucleus, PP2-A1:GFP fluorescence was found in the cytoplasm and in the nucleus (Fig. 7 A), as in companion cells. In differentiating cells displaying plastid autofluorescence but with no observable nucleus, PP2-A1:GFP continued to accumulate in the cytosol (Fig. 7 B). At later stages, in cells retaining large organelles, presumably lytic vacuoles, but no longer displaying plastid autofluorescence and nuclear staining, PP2-A1:GFP was observed both in the cytosol and as small dots of fluorescence, indicating the progressive anchoring of PP2-A1 to mitochondria or other organelles (Fig. 7 C-E). In mature sieve elements, the cytosolic fluorescence had completely disappeared, and PP2-A1:GFP appeared as discrete spots (Fig. 7 F).

## Discussion

### Live imaging of phloem cells in *Arabidopsis*

**Fig 8:**
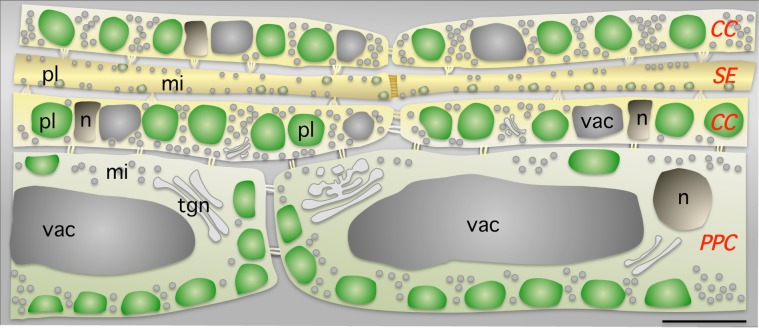
Organization of the phloem cells in mature *Arabidopsis* leaves. One straightforward criterion for distinguishing between phloem parenchyma cells (PPC), companion cells (CC) and sieve elements (SE) is the presence and positions of plastids (pl). In the CCs, plastids are aligned and occupy a large proportion of the cell volume. Numerous mitochondria (mi) are also found in each cell type. The nuclei (nu) and the vacuole (vac) have unusual shapes and positions in the CCs. Plastid size differs with cell type (1 μm in SE, 3 μm in CC and 4 μm in PPC), whereas mitochondrial size variations are more limited (0.6 μm in SE, 0.6 μm in CC and 0.7 μm in PPC).

Fluorescent markers were used for the *in vivo* imaging of subcellular components of phloem cells in detached *Arabidopsis* leaves (Fig. 8). Observations were made for a few minutes, immediately after sample preparation. SE observations on detached leaves may be problematic due to hydraulic disruption, as the pressure is likely to drop, making it difficult to refill the sieve tubes, in leaves disconnected from the plant xylem [33]. We carried out an experiment with CFDA to check that the transport activity of the phloem was preserved for several minutes. We also confirmed that leaf peeling did not substantially modify the subcellular organization of sieve elements, with respect to other methods, in experiments using fluorescently tagged proteins or vital markers [14, 34]. Virtually identical methods have been used on intact leaves from *Vicia faba* and tobacco, for the visualization of subcellular structures in phloem cells [17, 34]. However, transport in the phloem is an integrative phenomenon operating at whole-plant level that is dependent on sources, sinks, a transport tube and a source of water; live imaging results for phloem cells over more extended periods should therefore be interpreted with caution.

### Landmarks for the identification of companion cells and sieve elements

On longitudinal sections, the companion cells in leaf phloem present a distinctive feature, in the form of a remarkable single-file alignment of the chloroplasts (Fig. 8), which occupy a large proportion of the cell volume. As the chloroplasts and vacuoles of these cells take up most of their volume, the nucleus, mitochondria and ER are compacted, as observed by transmission electron microscopy [4, 35]. This situation is very different from that in phloem parenchyma cells, in which the distribution of chloroplasts at the cell periphery, opposite the companion cells, also constitutes a useful landmark for identification. Some of the fluorescent markers were not detected in mature sieve elements. Most organelles, such as nuclei and vacuoles, disintegrate during sieve element differentiation, and we would therefore not expect to observe them. Nevertheless, the membrane markers GFP:LTI6b (PM marker) and ER:YFP (ER marker), and the mitochondria COX4-GFP marker were observed. Either these markers diffused from the companion cell to the sieve element, or the fusion proteins were produced in differentiating sieve elements and were resistant to degradation in mature sieve tubes, unlike RuBisCO and histone 2B that are probably subjected to proteolysis during sieve element differentiation. By contrast, sieve element-resident proteins fused to GFP (GFP:RTM1, RTM2:GFP, SEOR1:GFP, SEOR2:GFP) produced under the control of their own promoters are evidently resistant to proteolysis and constitute reliable markers of sieve elements (Fig. 8). None of these fusion proteins diffused to adjacent cells, due to low solubility, anchorage to organelles or dimensions preventing passage through plasmodesmata due to the size exclusion limit.

### The cytoskeleton in phloem cells

The organization of the cortical microtubules and actin filaments was observed in the companion cells. The cortical microtubules displayed transverse, oblique or longitudinal orientations, with some heterogeneity between samples. F-actin was more difficult to visualize in the companion cells. Only longitudinal actin bundles and a few thin actin filaments were observed. In the sieve elements, no fluorescent signal was detected for F-actin or microtubule markers. This lack of signal may result from the inherent difficulty of observing thin cytoskeleton elements. As for some other GFP fusions, the fusion proteins may be too large or have an unsuitable chemical composition for passage through the pore-plasmodesmata units or another structure. Nonetheless, a recent study established the presence of a peripheral actin meshwork in the sieve elements of fava bean leaves, by immunocytochemistry and the use of the phalloidin fluorescent marker [26]. As far as cortical microtubules are concerned, small amounts of α-tubulin have been detected in the phloem sap exudate of pumpkin [36, 37], but β-tubulin is not detectable in the sap of most species [38] and there is currently no direct evidence for the assembly of microtubules in the sieve elements [2].

### Sieve elements: not so empty after all

The nature of many sieve element components remains unknown [39]. The use of fluorescent markers and fluorochromes facilitated the identification of some of the organelles in the sieve elements. For example, the filamentous structural P-proteins were found in a reticulate pattern, similar to that previously reported in roots [14, 15] for the sieve element reticulum, as reported for the sieve elements of tobacco [34]. There was also a large number of mitochondria, as reported in a previous study [4]. PP2-A1-GFP fluorescence was partially colocalized with mitochondria and with another class of organelles not stained with MitoTracker Red. A number of GFP-RTM1-labeled bodies, about 1 μm in diameter, were found evenly aligned along the sieve tubes. GFP-RTM1 did not colocalize with mitochondria, suggesting that at least some of these bodies may be plastids, the largest sieve element bodies other than mitochondria, with diameters of about 1 μm in *Arabidopsis*, as estimated by transmission electron microscopy [14, 28, 29]. RTM2:GFP fluorescence was found in a layer lining the plasma membrane that might be part of the parietal ground matrix. At several locations, it surrounded small round bodies at the periphery of the sieve elements. These organelles have yet to be identified, but small subcellular structures of similar diameters were also observed on transmission electron micrographs of the sieve elements (SE). Similar bodies have also been observed in the SE parietal ground matrix in *Arabidopsis* [14] and in potato SEs, in which it was suggested that they might be endocytotic vesicles [40].

The numbers and dimensions of bodies detected and stacked at the cell periphery or located in the lumen of sieve elements (up to 4 μm diameter in the leaf) also indicated that these cells might contain a large volume of organelles and protein bodies. These data are consistent with those recently reported for *Arabidopsis* and tobacco, in which an estimated 35% of the lumen is occupied by sieve tube constituents [14].

### PP2-A1:GFP as a marker for the live imaging of sieve elements

The events occurring during the maturation of sieve elements, a process taking only 16 to 21 hours, are difficult to study *in vivo*, because of the small proportion of differentiating sieve elements. Indeed, this task has been compared to searching for the proverbial needle in a haystack [1]. GFP-tagged PP2-A1 produced under the control of the SEOR2 promoter produced a bright fluorescent signal in immature sieve elements, making it possible to image differentiating sieve elements easily. In sieve elements in the early stages of differentiation, PP2-A1:GFP was found in the cytosol and nucleus. This distribution is similar to that observed in companion cells. At later stages, during the disappearance of the nucleus and the reorganization of the organelles, PP2-A1:GFP was partially soluble in the cytosol and began to aggregate around bodies at the cell periphery. In mature sieve elements, PP2-A1:GFP was no longer soluble and was associated exclusively with the surface of mitochondria and a second class of larger organelles. These patterns define three stages, paralleling those described in the phloem of the fixed root tissues from *Arabidopsis* [29], which could be used as a blueprint for *in vivo* studies on the differentiation of the sieve elements.

### Localization of PP2-A1:GFP in the sieve elements

Differentiating sieve elements undergo major intracellular reorganizations, to create an unobstructed lumen through the anchoring of the remaining organelles to the plasma membrane at the lateral borders [41]. This enables the sieve tubes to support mass flow and to compensate for the high pressure generated by sugar loading. Improvements in fixation methods for phloem cell observation by transmission electron microscopy (TEM) have led to the identification of minute clamp structures coupling the membranes of the mitochondria, sieve element reticulum and plasma membrane, in tomato, *Vicia fava* and, more recently, *Arabidopsis* [14, 41]. In *Arabidopsis*, TEM images have shown that the mitochondria and ER are covered with a halo of protein spikes forming the clamp structures and that these structures, together with vesicles, are embedded in an amorphous ground matrix forming a parietal layer [14]. The components of the parietal ground matrix and the clamps remain unknown. Immunolocalization studies have shown that PP2-A1 are located in the sieve elements at small, unidentified bodies, different from the P-proteins [31]. In mature sieve elements, PP2-A1:GFP was located at the periphery of mitochondria and of a second class of organelles with a diameter of about 1 μm. Immunogold labeling showed that PP2-A1 was associated with the sieve element plastids [31]. We therefore suggest that at least some of these bodies were plastids. In the parietal ground matrix, PP2-A1:GFP may thus be present in the clamp structures around mitochondria and plastids. However, the resolution obtained with CLSM was not sufficient to confirm whether it formed small protein spikes around the organelles, such as those observed by TEM and constituting the clamps [14].

### Functions of PP2-A1:GFP in phloem cells

PP2-A1 is synthesized in the companion cells (CC), before being translocated to sieve elements [19]. In CC, PP2-A1-GFP was observed in both the cytosol and the nucleus. A nucleocytoplasmic distribution has also been reported for a PP2-like protein in the mesophyll cells of *Nicotiana tabacum* [42]. This suggests that this chaperone [43], which has been shown to bind GlcNAc residues [44], may be involved in the nucleocytoplasmic shuttling of proteins modified by O-GlcNAcylation in CC cells, before their trafficking through the companion cell–sieve element plasmodesmata. Such a role has already been proposed for the tobacco phloem protein NCAPP1, which also binds to O-GlcNAc proteins [45]. The differential localization of PP2-A1, soluble in the cytoplasm and in the nucleus of companion cells and immature sieve elements, but aggregated in mature sieve elements, suggests that this protein is multifunctional. Multifunctionality is a basic feature of many protein-binding proteins involved in structure and signaling, such as Armadillo/β-catenin, which acts as a platform for many binding partners and is involved in both cell-to-cell junctions in animals and transcriptional regulatory processes [46]. PP2-A1 binds proteins in phloem exudate [44], and other PP2s have been shown to bind mRNAs [47, 48]. The PP2-associated structures found in the sieve elements may therefore retain proteins and RNAs within sieve elements, preventing their translocation by mass flow in functional sieve elements.

## Materials and Methods

### Plant materials and growth conditions

*Arabidopsis thaliana* (accession Columbia) plants were used for plant transformation and *in vivo* observations. Plants were grown in the greenhouse under long-day conditions (16 h light/8 h dark) in soil (Tref substrates or sand) and watered with Plant-Prod nutrient solution (Fertil-France, Boulogne Billancourt, France). The transgenic lines expressing subcellular markers are described in S2 Table. Crosses were carried out between homozygous plants.

### Expression vectors

The binary vectors encoding translational fluorescent fusion proteins (S1 Table) were constructed with the GATEWAY system (Invitrogen, Life Technologies, Saint-Aubin, France). The promoter regions of *AtSUC2* (1400 bp) and *AtSEOR2* (1000 bp) were amplified from genomic DNA extracted from *Arabidopsis* Col-O and inserted into pCR II TOPO (Invitrogen). The coding sequence of *AtPP2-A1* was amplified with the PP2A1*Not* and PP2A1*Xba*I primers (S3 Table) and inserted between the *Not*I and *Xba*I sites of pCR II TOPO. ATTB sites were created by PCR with the primers pSUC2attb1, pSEOR2attb1 and PP2A1attb2, and intact attB recombination sites were reconstituted with the primers attB1 and attB2. These PCR fragments were introduced into pDONR 207 (Invitrogen). The vectors for LR recombination are described in S1 Table. The constructs were verified by sequencing.

### Plant transformation

*Agrobacterium tumefaciens* C58pMP90 [49] was transformed with the binary vectors, by electroporation. For vectors derived from pGKG, we added the helper vector pSoup [50] to the transfection mixture. *Arabidopsis* plants were transformed by floral dipping [51]. Transformants were selected on kanamycin (50 mg l^-1^), hygromycin (15 mg l^-1^) or glufosinate (15 mg l^-1^). For each construct, at least eight primary transformants were screened and one line was chosen for further analysis on the basis of its fluorescence quality and lack of phenotypic alterations. Most observations were carried out on homozygous F3 plants.

### Cytology

Observations were performed on the veins of excised rosette or stem leaves of *Arabidopsis*. Clear images of the leaf vein system were obtained by using a razor blade to remove the overlying cell layers, corresponding to the epidermis and spongy mesophyll, from the abaxial side of the leaf, essentially as previously described [17]. Immediately after the cutting of the leaf petiole, the leaf was immersed in water, a small area (1 cm^2^) of the abaxial cell layers of the leaf was removed, and the leaf was rinsed and directly mounted in water or dye for imaging. Observations were carried out within a few minutes of preparation, by confocal laser scanning microscopy, and did not extend beyond 10 minutes after peeling. The clearest observations were obtained with leaves that were more than half-expanded, which should have gone through the sink-to-source transition [52]. For observations of mitochondria, samples were incubated for 5 min and mounted directly in 2.5 μg ml^-1^ MitoTracker Red CMXRos (Invitrogen) for observation. For live observations of nuclei in phloem cells, samples were mounted in 50 μg ml^-1^ propidium iodide (Molecular Probes). Observations on non-excised leaves from intact plants were performed on *Arabidopsis* seedlings grown *in vitro* for 15 days on vertical plates with a stereo fluorescence microscope. A rosette leaf was placed on a drop of water on a coverslip and the abaxial epidermis was removed with a razor blade. A second coverslip was added before fluorescence imaging with an Axiozoom V16 (Zeiss) microscope equipped with a Plan-Neofluar Z 2.3x/0.57 RWD 10.6 mm.

### Phloem loading assay

Phloem loading in leaves was monitored by gently dry-peeling away a small area (∼25 mm^2^) of the abaxial surface, at the leaf margin, with a razor blade, and immediately applying 5 μl of 5,6 carboxyfluorescein diacetate (CFDA)–mixed isomers (60 μg ml^-1^) (Invitrogen) to the exposed area. The treated leaves were observed immediately, under a Nikon SMZ-1500 binocular microscope equipped with a high-pressure mercury lamp (C-SHG1, Nikon) and a GFP filter, and the loading of the fluorescent dye was monitored by video recordings every 1.5 s, for 3 minutes. The dye progressed basipetally in the minor veins and then in higher order veins, over at least 3 minutes.

### Confocal laser scanning microscopy

At least 10 independent leaf samples were observed for each marker, and several sectors with phloem cells were observed per sample. For confocal laser scanning microscopy, leaves were mounted in water. Fluorescence images were obtained with either a Leica TCS-SP2-AOBS spectral confocal laser scanning microscope or a Leica SP5II-AOBS-Tandem HyD spectral confocal laser scanning microscope, both of which were equipped with a Leica HCX PL APO x 63 water immersion objective. CFP was excited with a 405 nm diode laser; GFP and chloroplasts were excited with the 488 nm line of the argon laser; YFP was excited with the 514 line of the argon laser, and RFP was excited with the 543 nm line of the He-Ne laser. Fluorescence emission was detected in the 450 to 520 nm range for the CFP construct, at 500 to 560 nm for GFP constructs, at 521 to 560 nm and 510 to 565 nm for SP2 and SP5, respectively, for YFP constructs, at 600 to 650 nm for MitoTracker Red, 610 to 650 nm for propidium iodide, 565 to 645 nm for RFP, and 684–784 nm and 645–738 nm, respectively, for SP2 and SP5, for chloroplast autofluorescence. Images were recorded and processed with LCS software version 2.5 (Leica Microsytems, Nanterre, France). CFP, YFP and GFP fluorescence data were acquired by sequential scanning, with the 405 nm laser diode used to excite CFP, the 488 nm laser line used to excite GFP and the 514 nm laser line used to excite YFP. All images were single confocal 512 x 512-pixel scans, except for images of the cytoskeleton (Z-stack of 6 images with a step of 0.32 μm) and mitochondria (Z-stack of 20 images with a step of 0.25 μm).

### Ultrastructure on transmission electron microscopy

Small main vein or petiole sections were fixed by incubation, under vacuum, with 2.5% glutaraldehyde in 0.1 M phosphate buffer (pH 7.2) supplemented with 0.25 M sucrose for 3 h at 4°C. They were post-fixed by incubation with 1% osmium tetroxide in the same buffer for 2 h at 4°C. They were then dehydrated in a graded series of ethanol concentrations (30–100%), subjected to propylene oxide substitution, infiltrated and embedded in Epon resin. Ultrathin sections (50 nm) were cut on a Leica Ultracut-E microtome, stained with 7% uranyl acetate for 15 minutes and then with lead citrate for 5 minutes. Sections were examined at 80 kV in a FEI CM-10 transmission electron microscope equipped with an X-60 AMT digital camera (Elexience, Verrières-le-Buisson, France).

### Image analysis

The size of the protein bodies observed by CLSM was analyzed with the NIH image analysis program ImageJ v1.43u (NIH, http://rsb.info.nih.gov/ij/). Plastid size was analyzed by using ImageJ for measurements on images of plastid autofluorescence. Similarly, TEM images were analyzed to determine the size of plastids and mitochondria in the sieve elements.

## Supporting Information

S1 Fig Observation of attached leaves with a stereo fluorescence microscope. (TIF)

S2 Fig Imaging of fluorescent markers in phloem cells. (TIF)

S3 Fig Dimensions of the plastids in different cell types in the leaf. (TIF)

S4 Fig Imaging of fluorescent proteins in phloem cells. (TIF)

S5 Fig Unknown bodies observed by TEM in the sieve elements. (TIF)

S6 Fig Imaging of PP2-A1 in the sieve elements. (TIF)

S1 Movie Transport of carboxyfluorescein in the phloem. The CFDA is loaded in the phloem and the CF moves toward the petiole. The bright area is the treated area observed in Fig. 1 C.
(AVI)

S2 Movie 3D view of mitochondrial organization in phloem cells. Observation of mitochondria in the phloem cells of a *p35S*:*COX4*:*GFP* plant. GFP fluorescence is presented in false color yellow (left panel) and autofluorescence of the chloroplasts in false color red. The movie represents a 3D reconstruction of a Z-stack of 20 images with a step of 0.25 μm.
(MOV)

S1 Table Transgenic lines producing fluorescent proteins in phloem cells. (DOCX)

S2 Table Transgenic lines producing fluorescent proteins used for crosses. (DOCX)

S3 Table Description of the primers used for cloning promoters and coding sequences used in the expression vectors. (DOCX)
