# Supplementary figures and images for: Live Imaging of Companion Cells and Sieve Elements in Arabidopsis Leaves
Source: PLoS One. 2015 Feb 25;10(2):e0118122. doi: 10.1371/journal.pone.0118122 (PMC4340910; doi:10.1371/journal.pone.0118122)

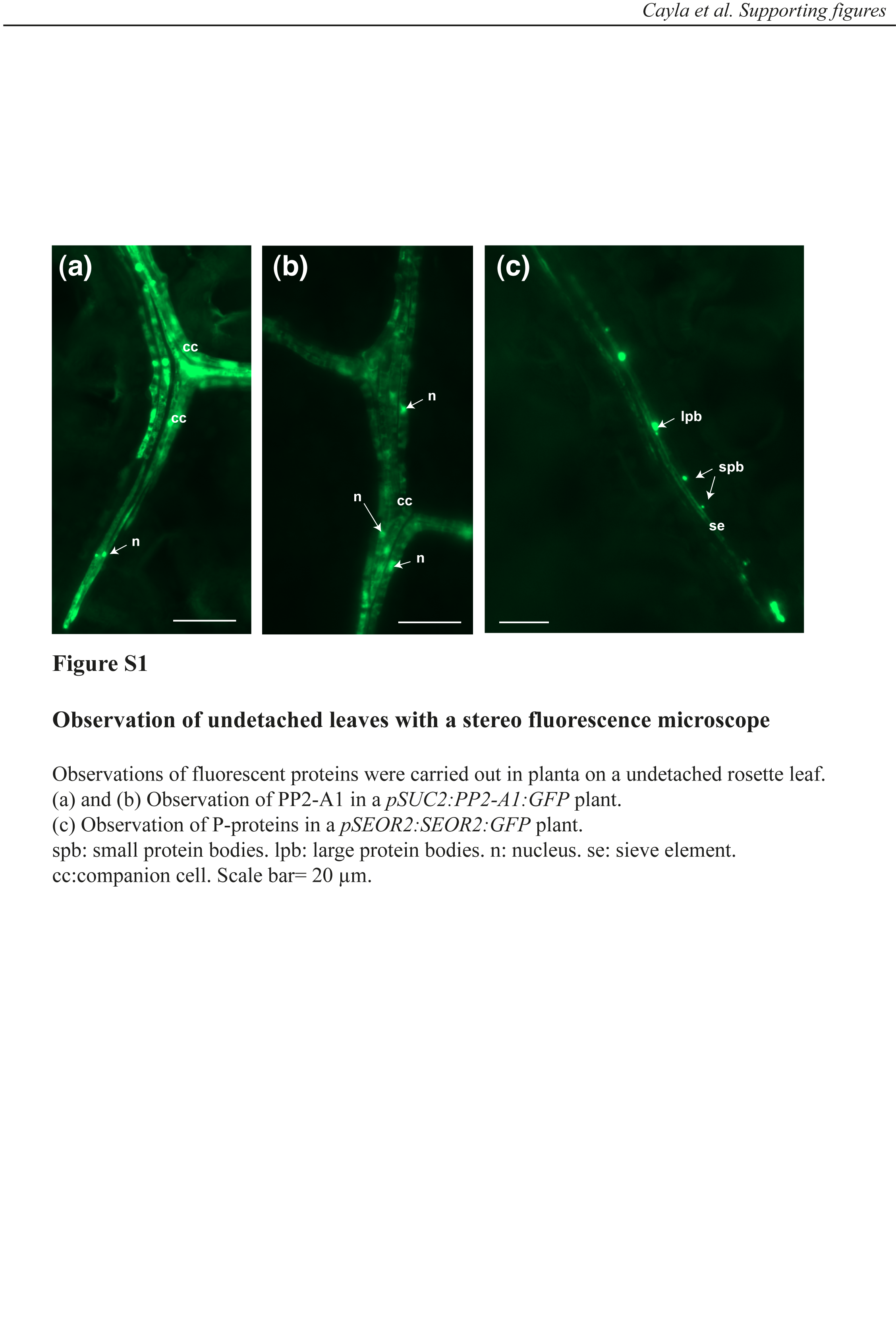

Supplement: S1 Fig — (TIF) [file pone.0118122.s001.tif]

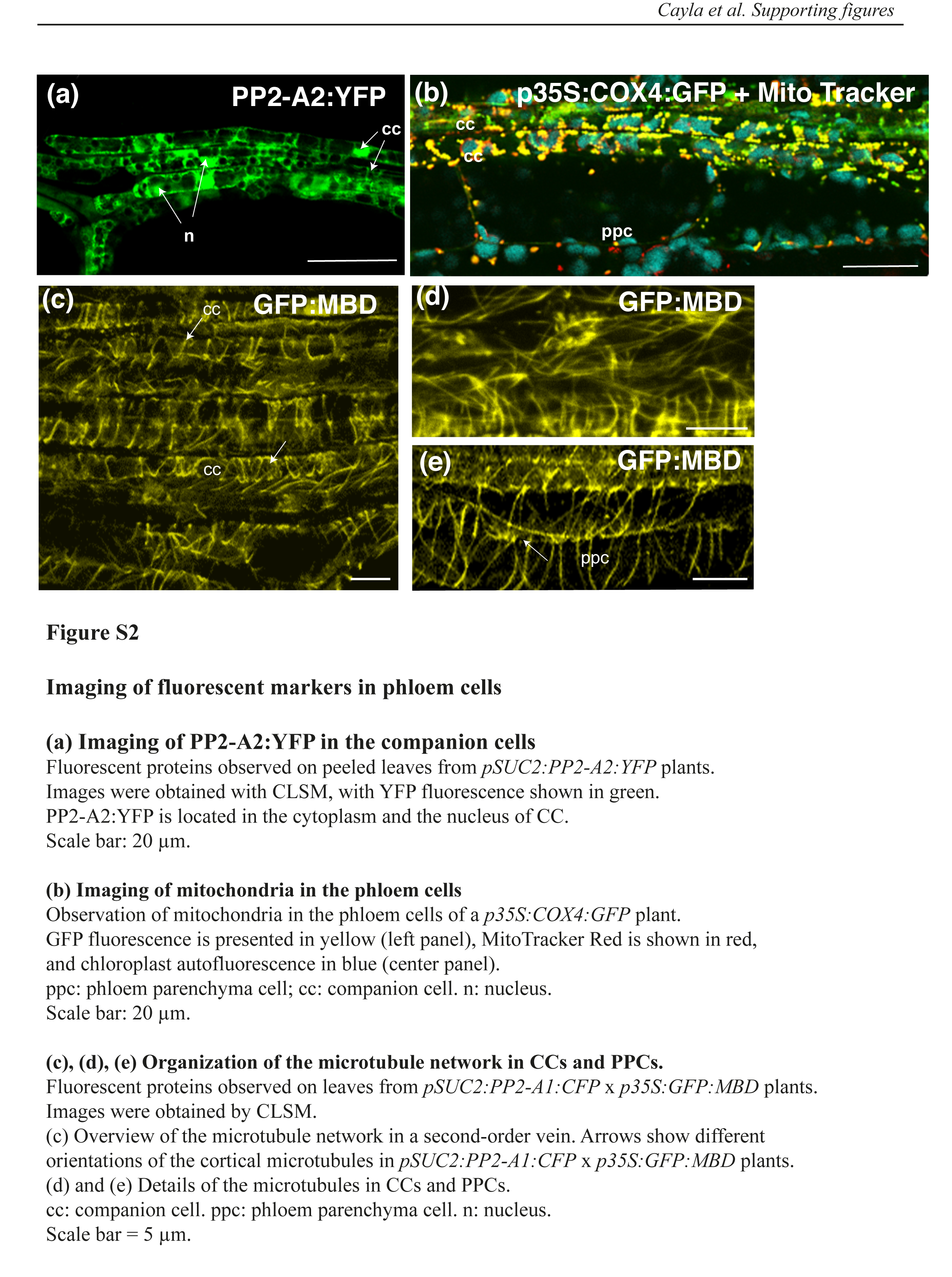

Supplement: S2 Fig — (TIF) [file pone.0118122.s002.tif]

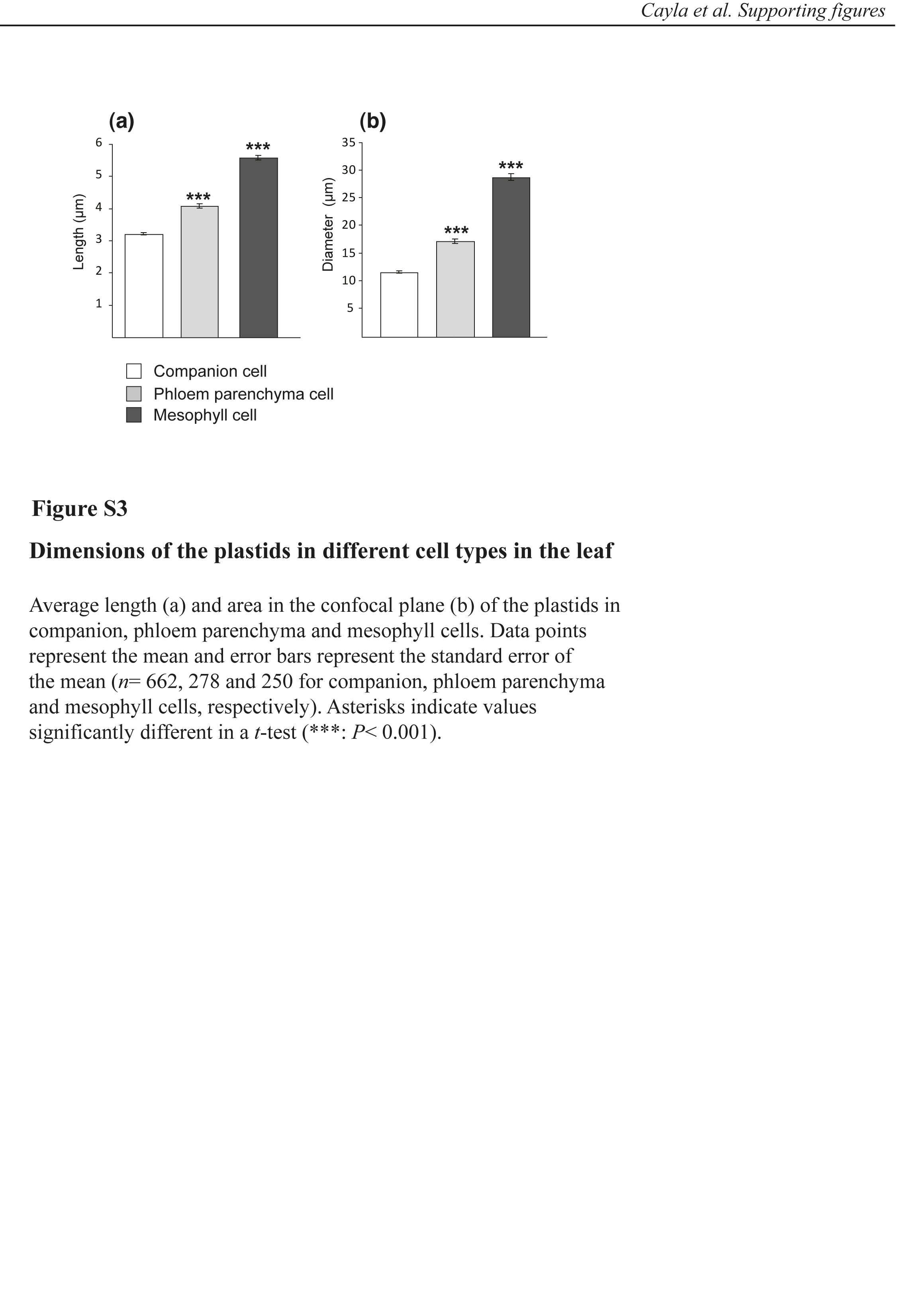

Supplement: S3 Fig — (TIF) [file pone.0118122.s003.tif]

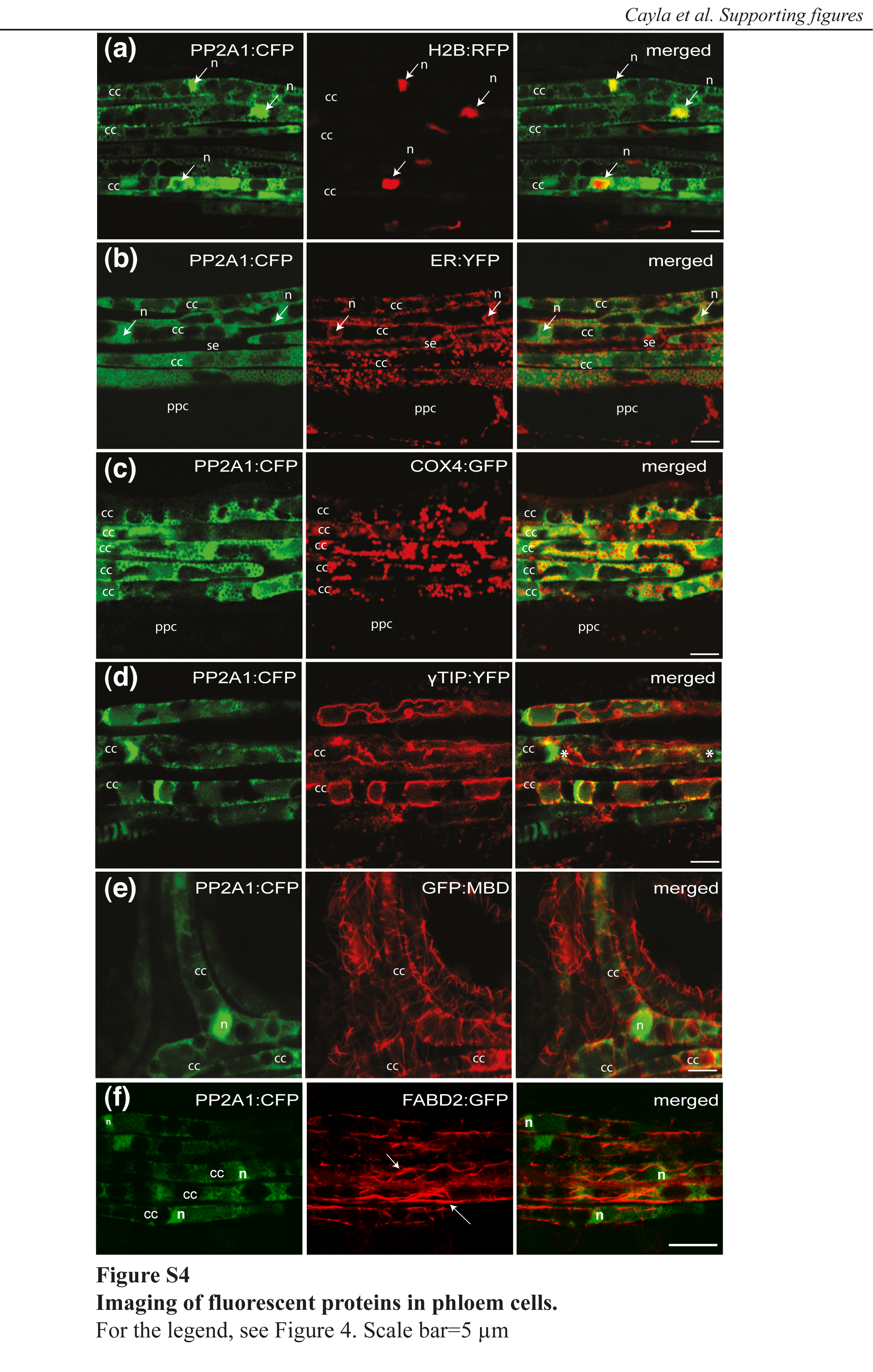

Supplement: S4 Fig — (TIF) [file pone.0118122.s004.tif]

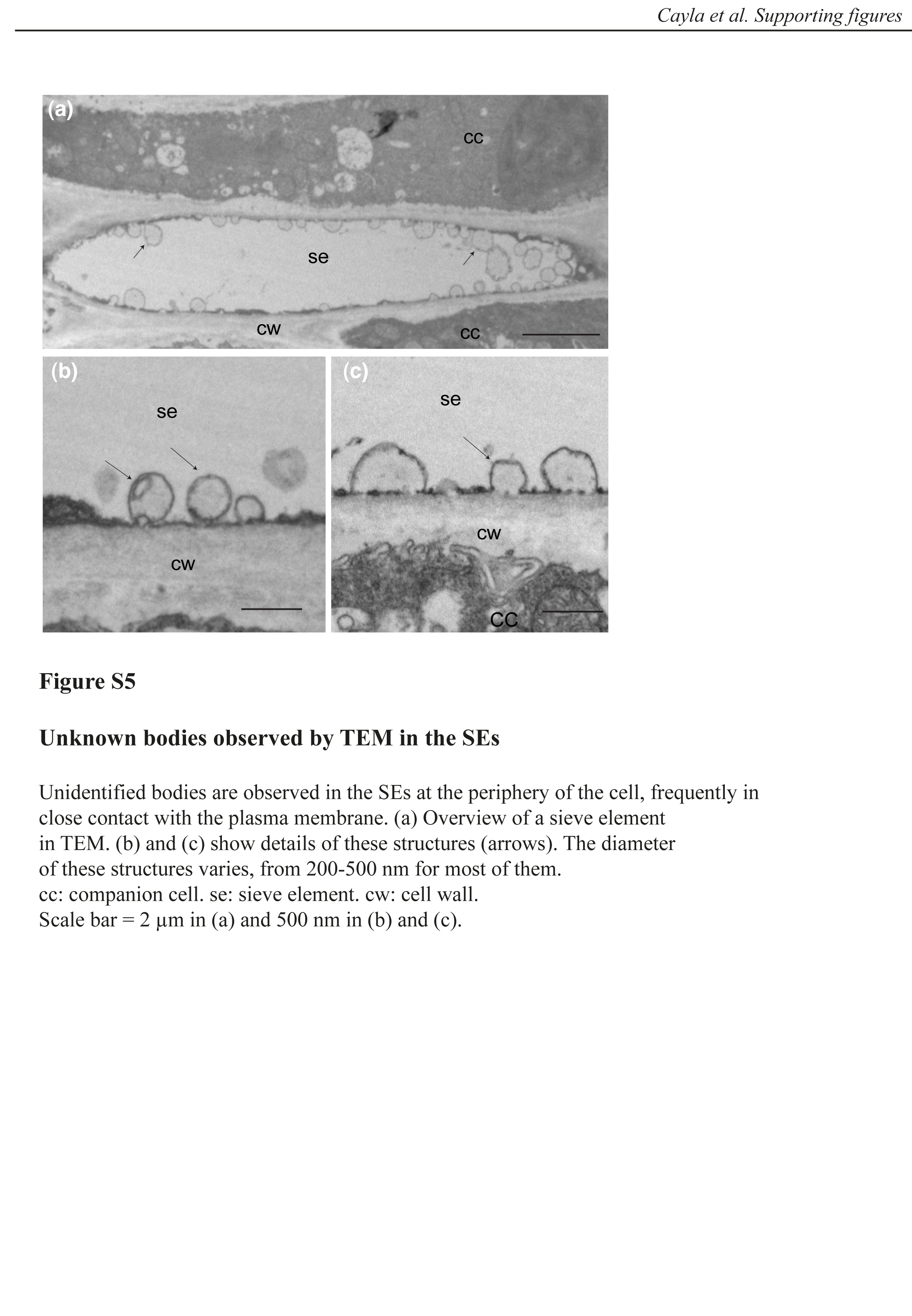

Supplement: S5 Fig — (TIF) [file pone.0118122.s005.tif]

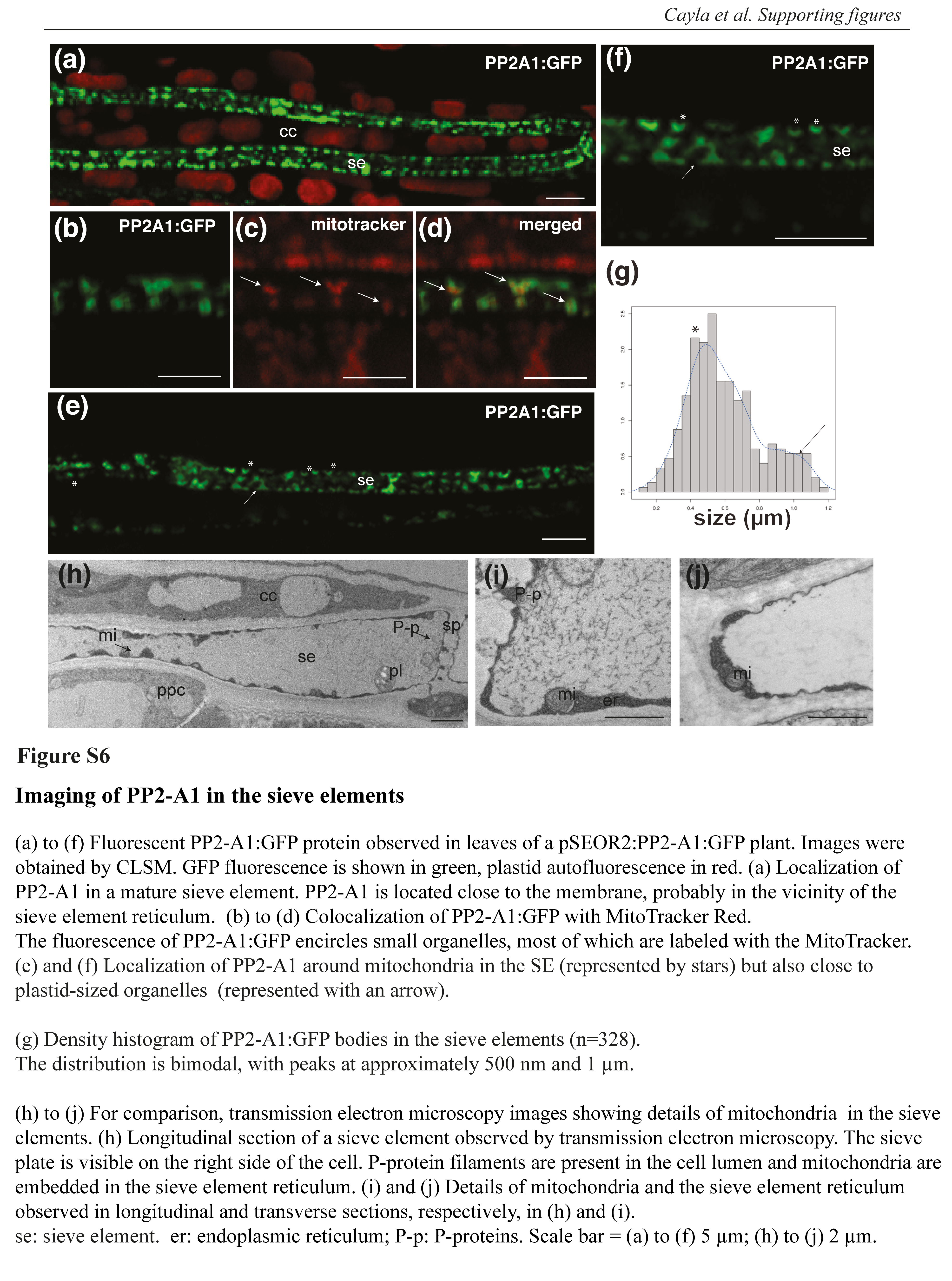

Supplement: S6 Fig — (TIF) [file pone.0118122.s006.tif]
