# Supplementary material for: Live Imaging of Companion Cells and Sieve Elements in Arabidopsis Leaves
Source: PLoS One. 2015 Feb 25;10(2):e0118122. doi: 10.1371/journal.pone.0118122 (PMC4340910; doi:10.1371/journal.pone.0118122)
Supplement: S1 Table — (DOCX) [file pone.0118122.s009.docx]

*Cayla et al. Supporting tables*

**S1_ Table: Transgenic lines expressing fluorescent proteins in phloem cells**

| **Construct** | **Number of primary transformants** | **Destination vector** |
| --- | --- | --- |
| *pSUC2:GFP* | 13 | pMDC107 [1] |
| *pSUC2:PP2-A1:GFP* | 9 | pGKGWG [2] |
| *pSUC2:PP2-A1:CFP* | 15 | pGBGWC [2] |
| *pSUC2:GFP:PP2-A1* | 8 | pGWB1 [3] |
| *pSUC2:PP2-A2:YFP* | 2 | pGKGWY [2] |
| *pSEOR2:PP2-A1:GFP* | 7 | pGKGWG [2] |

**Références**

1. Curtis MD, Grossniklaus U (2003) A Gateway cloning vector set for high-throughput functional analysis of genes *in planta*. Plant Physiology 133: 462-469.

2. Zhong S, Lin Z, Fray R, Grierson D (2008) Improved plant transformation vectors for fluorescent protein tagging. Transgenic Research 17: 985-989.

3. Nakagawa T, Kurose T, Hino T, Tanaka K, Kawamukai M, Niwa Y, Toyooka K, Matsuoka K, Jinbo T, Kimura T (2007) Development of series of gateway binary vectors, pGWBs, for realizing efficient construction of fusion genes for plant transformation. Journal of Bioscience and Bioengineering 104: 34-41.
