# Supplementary material for: Live Imaging of Companion Cells and Sieve Elements in Arabidopsis Leaves
Source: PLoS One. 2015 Feb 25;10(2):e0118122. doi: 10.1371/journal.pone.0118122 (PMC4340910; doi:10.1371/journal.pone.0118122)
Supplement: S2 Table — (DOCX) [file pone.0118122.s010.docx]

*Cayla et al. Supporting tables*

**S2_Table: Transgenic lines expressing fluorescent proteins used for crosses**

**Gene accession numbers:** The AGI numbers of the genes used in this work are for *PP2-A1*: At4g19840; *PP2-A2*: At4g19850, *SEOR1*: At3g01680; *SEOR2*: At3g01670; *SUC2*: At1g22710, *RTM1*: At1g05760; *RTM2*: At5g04890; *Lti6B*: At3g05890.

| **Line** | **Marker** | **Description** | **Reference** |
| --- | --- | --- | --- |
| *p35S:ER:YFP* | Endoplasmic reticulum | Signal peptide of AtWAK2 and ER retention signal HDEL | [1] |
| *p35S:RbcS:YFP* | Chloroplast | First 79 aa of the tobacco small unit of RuBisCO | [1] |
| *p35S:GFP:MBD* | Cortical microtubule | Microtubule binding domain | [2] |
| *p35S:fABD2:GFP* | Actin filament | Actin binding domain fABD2 | [3] |
| *p35S:γTIP:YFP* | Tonoplast | C-terminus of the tonoplast γTIP | [1] |
| *p35S:H2B:RFP* | Nucleus | Human histone H2B | [4] |
| *pSUC2:mcitrine:RCI2A* | Membranes | RCI2A full-length (low-temperature-inducible protein a) | [5] |
| *p35S:GFP:LTI6b* | Plasma membrane | LTI6b protein (low temperature-inducible protein b), also known as RCI2bRCI2B | [6] |
| *p35S:COX4:YFP* | Mitochondria | First 29 aa of yeast ScCOX4 | [1] |
| *pSEOR1:SEOR1:GFP* | P-protein | SEOR1, full-length | [7] |
| *pSEOR2:SEOR2:GFP* | P-protein | SEOR2, full-length | [7] |
| *pRTM1:GFP:RTM1* | - | RTM1, full-length | [8] |
| *pRTM2:RTM2:GFP* | - | RTM2, full-length | [8] |

**Références**

1. Nelson BK, Cai X, Nebenführ A (2007) A multicolored set of *in vivo* organelle markers for co-localization studies in *Arabidopsis* and other plants. Plant Journal 51: 1126-1136.

2. Camilleri C, Azimzadeh J, Pastuglia M, Bellini C, Grandjean O, Bouchez D (2002) The *Arabidopsis* TONNEAU2 gene encodes a putative novel protein phosphatase 2A regulatory subunit essential for the control of the cortical cytoskeleton. The Plant Cell 14: 833-845.

3. Voigt B, Timmers AC, Samaj J, Muller J, Baluska F, Menzel D (2005) GFP-fABD2 fusion construct allows *in vivo* visualization of the dynamic actin cytoskeleton in all cells of *Arabidopsis* seedlings. Eur J Cell Biol 84: 595-608.

4. Boisnard-Lorig C, Colon-Carmona A, Bauch M, Hodge S, Doerner P, Bancharel E, Dumas C, Haseloff J, Berger F (2001) Dynamic analyses of the expression of the histone::YFP fusion protein in *Arabidopsis* show that syncytial endosperm is divided in mitotic domains. The Plant Cell 13: 495-509.

5. Thompson MV, Wolniak SM (2008) A plasma membrane-anchored fluorescent protein fusion illuminates sieve element plasma membranes in *Arabidopsis thaliana* and *Nicotiana tabacum*. Plant Physiology 146: 1599-1610.

6. Cutler SR, Ehrhardt DW, Griffitts JS, Somerville CR (2000) Random GFP∷cDNA fusions enable visualization of subcellular structures in cells of *Arabidopsis* at a high frequency. Proceedings of the National Academy of Sciences 97: 3718-3723.

7. Anstead JA, Froelich DR, Knoblauch M, Thompson GA (2012) *Arabidopsis* P-Protein filament formation requires both AtSEOR1 and AtSEOR2. Plant and Cell Physiology 53: 1033-1042.

8. Chisholm ST, Parra MA, Anderberg RJ, Carrington JC (2001) *Arabidopsis* *RTM1* and *RTM2* genes function in phloem to restrict long-distance movement of tobacco etch virus. Plant Physiology 127: 1667-1675.
