# Supplementary material for: Live Imaging of Companion Cells and Sieve Elements in Arabidopsis Leaves
Source: PLoS One. 2015 Feb 25;10(2):e0118122. doi: 10.1371/journal.pone.0118122 (PMC4340910; doi:10.1371/journal.pone.0118122)
Supplement: S3 Table — (DOCX) [file pone.0118122.s011.docx]

*Cayla et al. Supporting tables*

**S3_ Table: Description of the primers used for cloning promoters and coding sequences used in the expression vectors**

| **Primer name** | **Sequence** |
| --- | --- |
| AttB1 | 5’- ggggacaagtttgtacaaaaaagcaggct –3’ |
| AttB2 | 5’– ggggaccactttgtacaagaaagctgggt –3’ |
| PP2A1attb2 | 5’– aagaaagctgggtgctgtttgggacgaattgc –3’ |
| PP2A1Not | 5’– tggcggccgcatgtttagaaaccaagac –3’ |
| PP2A1XbaI | 5’– cctctagactgtttgggacgaattgc –3’ |
| pSEOR2-fw | 5’ – gctactcagcccggagatcatccggcaa –3’ |
| pSEOR2-rv | 5’ – gcattggcgaggttgagaaatatatg – 3’ |
| pSEOR2attb1 | 5’– aaaaagcaggctgctactcagccggagatcat –3’ |
| pSUC2-fw | 5’ – ctcaagtgtttcttttttaagg – 3’ |
| pSUC2-rv | 5’ – atttgacaaaccaagaaagtaa – 3’ |
| pSUC2SalI-fw | 5’– gggagctctcaatgaaatcccatagtag –3’ |
| pSUC2Xho-rv | 5’– gggtcgagatggtcagccatccaatg –3’ |
| pSUC2attb1 | 5’– aaaaaagcaggcgatagttaattatttggggg –3’ |
| pSUC2FWpcr | 5’–cttaacttcactgtctcctc –3’ |
